# The nCOVID-19 and financial stress in the USA: health is wealth

**DOI:** 10.1007/s10668-020-01029-w

**Published:** 2020-10-08

**Authors:** Andrew Adewale Alola, Uju Violet Alola, Samuel Asumadu Sarkodie

**Affiliations:** 1 Department of Economics and Finance, Istanbul Gelisim University, Istanbul, Turkey; 2 Department of Financial Technologies, South Ural State University, Chelyabinsk, Russia; 3 Department of Tourism Guidance, Istanbul Gelisim University, Istanbul, Turkey; 4 Economics and Management, South Ural State University, Chelyabinsk, Russia; 5 Nord University Business School (HHN), Post Box 1490, 8049 Bodø, Norway

**Keywords:** COVID-19 pandemic, Daily deaths, Daily recoveries, Economic uncertainty, Financial stress, USA, C41, G41, I1, I120, I150

## Abstract

Since its first report in the USA on 13 January 2020, the novel coronavirus (nCOVID-19) pandemic like in other previous epicentres in India, Brazil, China, Italy, Spain, UK, and France has until now hampered economic activities and financial markets. To offer one of the first empirical insights into the economic/financial effect of the COVID-19 pandemic, especially in the USA, this study utilized the daily frequency data for the period 25 February 2020–30 March 2020. By employing the empirical Markov switching regression approach and the compliments of cointegration techniques, the study establishes a two-state (stable and distressing) financial stress situation resulting from the effects of COVID-19 daily deaths, COVID-19 daily recovery, and the USA’ economic policy uncertainty. From the result, it is assertive that daily recovery from COVID-19 eases financial stress, while the reported daily deaths from COVID-19 further hamper financial stress in the country. Moreover, the uncertainty of the USA’ economic policy has also cost the Americans more financial stress and other socio-economic challenges. While the cure for COVID-19 remains elusive, as a policy instrument, the USA and similar countries with high severity of COVID-19 causalities may intensify and sustain the concerted efforts targeted at attaining a landmark recovery rate.

## Introduction

**Fig. 1:**
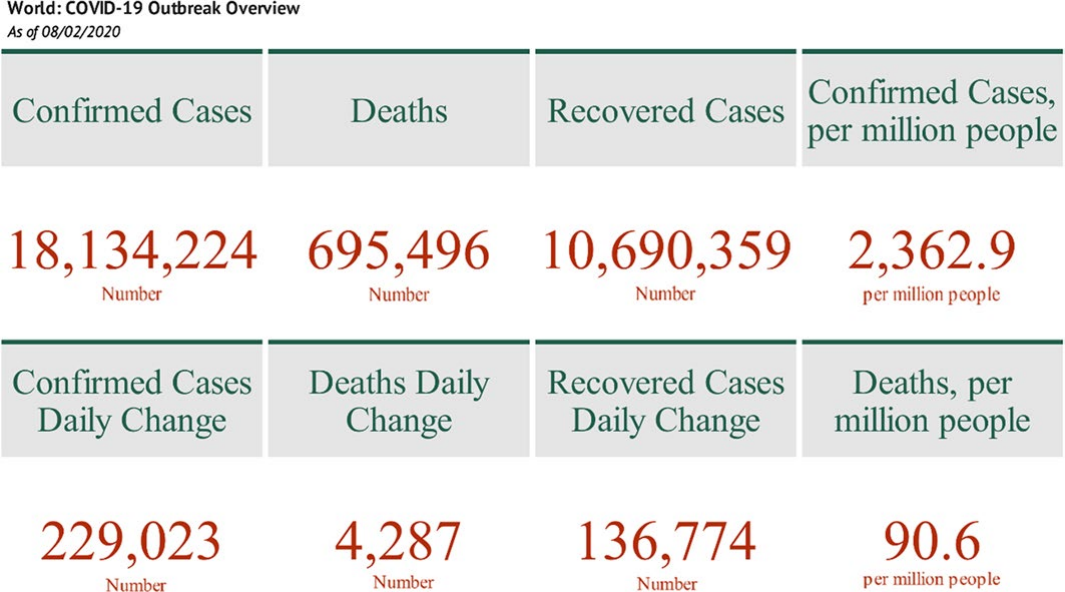
Global COVID-19 pandemic overview. Data: Lauren (2020)

With 32,429,965 cases (consisting of over 200 countries and territories) and accounting for the death of 985,823 persons globally as reported by the World Health Organization (WHO 2020) on 26 September 2020, the ravaging effect of the novel coronavirus disease (COVID-19) has remained a global emergency. As of August 2 2020, there were 18,134,224 reported confirmed cases, 695,496 deaths, and 10,690,359 recovered cases—corresponding to 229,023 daily change in confirmed cases, 4287 daily change in deaths, and 136,774 daily change in recovery cases [see Fig. 1] (Lauren 2020). So far, the dreadfulness of the COVID-19 pandemic has further irked global reflection and the imagination of the world’s most devastating epidemic, the influenza pandemic or ‘Spanish Flu’ of 1918–1919 (Stanford University 2005). Since the January 30, 2020 declaration of the COVID-19 as a global emergency by WHO and the subsequent global determination to ‘flatten the curve’, a few outlined measures have been implemented. Some of the measures that are currently in place or now gradually have been suspended across the governments include the ‘lockdown’, ‘social distancing’, travel restriction/suspension between countries, quarantine, and other measures differ across states (Sarkodie and Owusu 2020a). Giving the implementation of these measures, most nations continue to experience a spillover effect of the COVID-19 pandemic to the socio-economic aspects: the primary sectors, secondary sectors, and the tertiary sectors of the world economy (Kostova et al. 2019; Gregori et al. 2020; Khalatbari-Soltani et al. 2020; Mason-D’Croz et al. 2020; Nicola et al. 2020). To safeguard livelihoods and sustain economic development due to the global pandemic shocks, several economic activities instituted across countries include fiscal policy cut, exchange rate, and monetary intervention (Sarkodie and Owusu 2020b).

Importantly, the aspects of the world’s economy, especially that of the USA, and other leading economies such as China, UK, Germany, France, Italy, Japan, and Spain, are fast becoming the shadow of its image. For instance, the USA (as the largest economy by the measure of gross domestic product, GDP), has suffered the worst unemployment crisis since the Great Depression. With 7,078,798 reported cases, 204,497 human deaths from COVID-19, a record high of 14.7% unemployment rate by April 2020, and the massive oil price slump in the USA (Financial Times 2020; Johns Hopkins University and Medicine 2020; United States Bureau of Labour Statistics 2020), the effect of COVID-19 pandemic continues to hamper every aspect of the country’s economy. In response to the USA’ West Texas Intermediate (WTI), record oil price collapse (falling below 0 $) amidst an increasing decline in demand arising from the COVID-19 pandemic, the country’s government has twice provided stimulus packages to alleviate the financial distress among the Americans. The stimulus package as contained in the S.3548—CARES Act is expected to help businesses, families, and the individuals affected by the 2020 coronavirus pandemic with emergency assistance and health care response (United States Congress 2020), thus alleviating financial stress. Notwithstanding, these efforts of the US government are insufficient at addressing the argument that has consistently corroborated the link between high infection and mortality of COVID-19 among the African American and Latino population with the socio-economic disadvantage of these racial groups (Abedi et al. 2020; de León-Martínez et al. 2020).

**Fig. 2:**
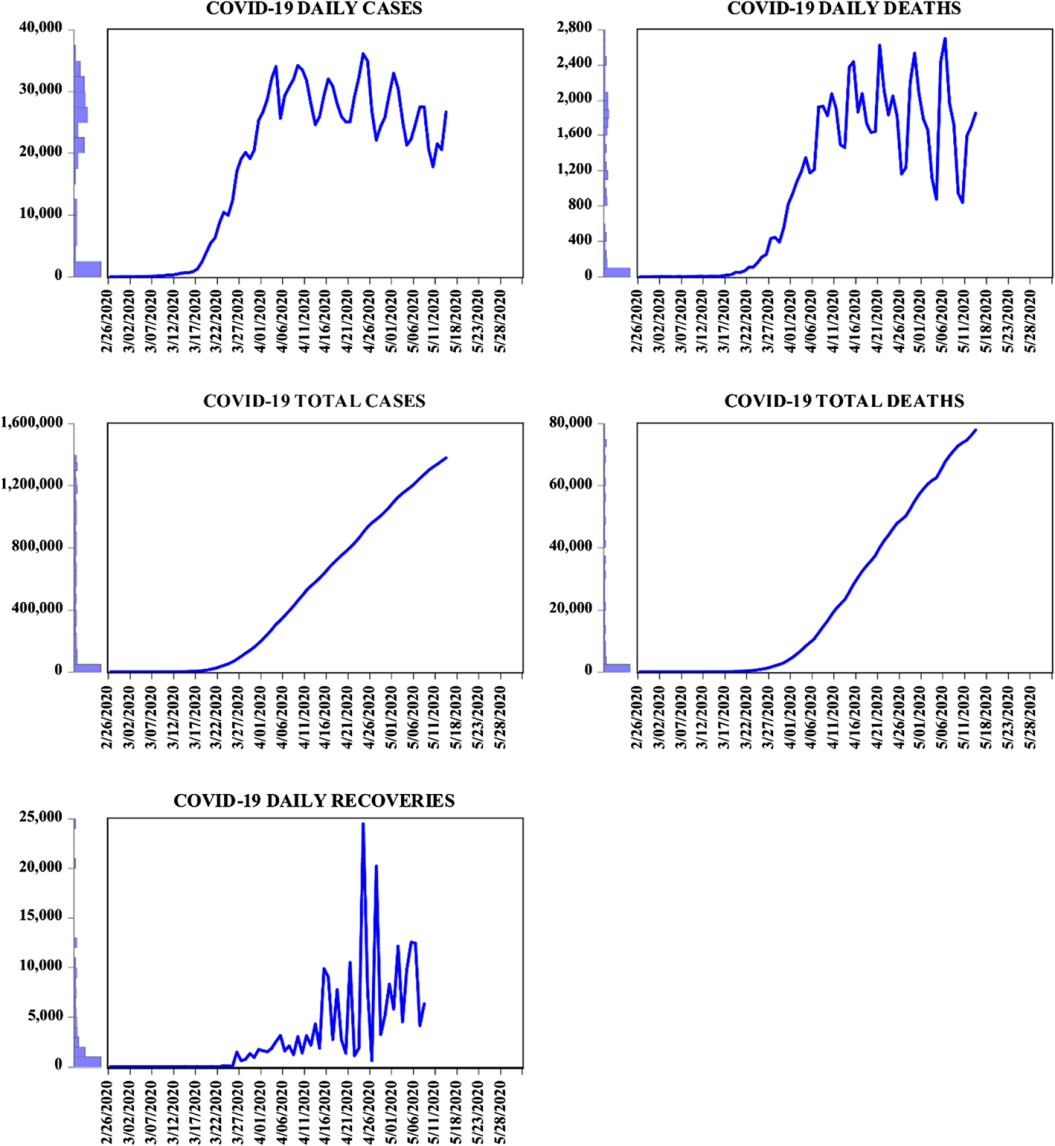
The line plot for COVID-19 for daily cases, daily deaths, total cases, total deaths, and daily recoveries in the USA. (Data are computed from Johns Hopkins University and Medicine and Centers for Diseases Control and Prevention (CDC 2020)

Considering the above motivation, this concise study is billed at illustrating the financial stress consequence of the ravaging COVID-19 pandemic in the USA. Giving the almost unpredictability of the later cases of COVID-19 deaths and recoveries as against that of the total COVID-19 cases and deaths as illustrated in Fig. 2, the current study seeks to establish a link between the health emergencies arising from COVID-19 and financial stress. Giving that only a sparse study such as Nicola et al (2020) has explored the economic aspects of COVID-19, the novelty of the current study is expected to close the existing gap in the literature through the following approaches: (i) the examining of the impact of daily deaths from COVID-19, daily recoveries from COVID-19, and economic uncertainty on financial stress, (ii) illustrating with empirical evidence of the states of financial stress with the regime switching approach of Markov-Switch regression technique, and (iii) the use of daily frequency and quite up-to-date data spanning the period 25 February 2020–30 March 2020. With the aforementioned novel approaches, this study is capable of further asserting that indeed ‘health is wealth’.

In the succeeding part of this study, the material employed, theoretical concept, the discussion of the results, and the conclusion of the study are all outlined orderly in sections two, section three, section four, and section five, respectively.

## Materials and method

In the USA and across major economies of the world, nothing else could be imagined possessing the potential of disrupting the normal economic activities and financial markets like the COVID-19 pandemic. Having caused the closure or suspension of international air travels, disruption of energy production and especially the oil prices, disruption of agricultural activities, closure of school activities at all levels and other disruptions, the daily deaths and recoveries from COVID-19 in the USA have since compounded the Americans financial woes. The resulting disruption of normal economic activities and financial markets is akin to the country’s prevailing financial stress (Hakkio and Keeton 2009).

Thus, in examining the probable financial stress experienced in the USA due to the COVID-19 pandemic, the following materials are employed:

- Since the first reported (three) cases of nCOVID-19 in the USA by the CDC (2020), the data for the daily deaths (DD) and recoveries (RC) from the nCOVID-19 disease are provided by the Johns Hopkins University and Medicine (2020).
- The indexes of financial stress and economic policy uncertainty of the US Economic Policy Uncertainty^1^ are used as proxies to capture financial stress (FS) and economic policy (EU), respectively. Because of restricted data availability, the series is considered for the daily frequency period of 25 February 2020–30 March 2020.

Consequently, the method employed in the study is based on the preliminary findings of the statistical evidence of correlation (see Table 1) among the aforementioned factors and the individual statistical properties (see Table 2). Importantly, the data show the high variance of death and recoveries from COVID-19 as well as the economic uncertainty.

**Table 1:** Correlation among the varying factors

Variable	FS	DD	RC	EU
FS	1.000			
DD	0.649^a^	1.000		
	(0.000)	–		
RC	0.492^a^	0.814^a^	1.000	
	(0.003)	(0.000)	–	
EU	0.914^a^	0.580^a^	0.535^a^	1.000
	(0.000)	(0.000)	(0.001)	–

The varying factors FS, EU, DD, and RC are, respectively, the financial stress, economic uncertainty, daily deaths from coronavirus (COVID-19), and the daily recoveries from COVID-19 disease in the USA. The estimation shows that there is 1% (indicated as^a^) statistically significant correlation among the factors

**Table 2:** Common statistics of the varying factors

Statistic	DD	RC	FS	EU
Mean	85.0857	157.3143	2.5943	343.1240
SD	150.5846	380.7394	2.1233	193.4675
Variance	22,675.7277	144,962.5160	4.5086	37,429.6570
Skewness	1.9871	2.5854	0.0955	0.4482
Kurtosis	2.9410	5.7794	− 1.5571	− 1.1327
Coefficient of variation	1.7698	2.4202	0.8185	0.5638
Minimum	0	0	− 0.5841	97.49
1st Quartile (Q1)	3	0	0.5486	172.45
Median	7	2	3.2857	296.12
3rd Quartile (Q3)	109	33	4.956	515.67
Maximum	554	1470	5.3736	743.24
Jarque–Bera test	29.2740	69.5550	3.3740	2.9810
Probability	0.0000	0.0000	0.1850	0.2250
*N* total	35	35	35	35

The varying factors FS, EU, DD, and RC are, respectively, the financial stress, economic uncertainty, daily deaths from coronavirus (COVID-19), and the daily recoveries from COVID-19 disease in the USA

### Theoretical and empirical concept

Nicola et al (2020) provided a foundation for the economic and financial perspective of nCOVID-19. However, Park and Mercado (2014) illustrated the empirical relationship between financial stress and economic uncertainty. By extending this concept, the financial stress (FS) effect of nCOVID-19 amidst economic uncertainty according to Nicola et al (2020) can be illustrated in an ordinary least square (OLS) framework as:

1 $${\text{Financial Stress}}_{t} = c_{0} + c_{1} {\text{COVID }} - {\text{ 19 Daily Deaths}}_{t} + c_{2} {\text{COVID}} - {\text{19 Daily Recoveries}}_{t} + c_{3} {\text{EU}}_{t} +\upvarepsilon _{t}$$

But, by incorporates the switching parameter as regressors, the approach of Markov switching regression from the work of Hamilton (1989) and Reboredo (2010) is applied to the current concept through the following:

2 $${\text{FS}}_{i,t} = \kappa_{0,i,rt} + \kappa_{1,i,rt} {\text{DD}}_{t} + \kappa_{2,i,rt} {\text{RC}}_{t} + \kappa_{3,i,rt} {\text{EU}}_{t} + {\upvarepsilon }_{i,t}$$

Given that for all ${\upvarepsilon }_{{\text{i,t}}}$ ~ N (0, σst2), the variance of the error is where *i* is the (35) daily period starting from 25 February 2020 to 30 March 2020 for USA’ COVID-19 daily deaths (DD), COVID-19 daily recoveries (RC), daily economic uncertainty (EU). Therefore, the impact of DD, RC, and EU on financial stress (FS) is, respectively, $\kappa_{{1,{\text{i}},{\text{rt}}}} , \kappa_{{2,{\text{i}},{\text{rt}}}} , \kappa_{{3,{\text{i}},{\text{rt}}}}$. A latent unobserved state variable *st* is the two regimes 1 and 2 that represents a more ***stable*** and a ***distressing*** financial stress regime, respectively. In this context, the transition probability of the estimation matrix is given as:

3 $$P(t) = \left[ {\begin{array}{*{20}l} {P_{t} 11} \hfill & {1 - P_{t} 12} \hfill \\ {1 - P_{t} 21} \hfill & {P_{t} 22} \hfill \\ \end{array} } \right]$$

And, given the dynamic nature of the DD, RC, and EU, especially in the current circumstance of COVID-19 in the USA, the aforementioned probabilities of the transitioning states are given as:

4a $$\mathop {P11}\nolimits_{t} = \frac{{\exp \left\{ {\mathop \gamma \nolimits_{1} + \mathop x\nolimits_{1} \mathop p\nolimits_{t - 1}^{{{\text{DD}}}} + \mathop u\nolimits_{2} \mathop q\nolimits_{t - 1}^{{{\text{RC}}}} + \mathop v\nolimits_{1} \mathop r\nolimits_{t - 1}^{{{\text{EU}}}} } \right.}}{{1 + \exp \left\{ {\mathop \gamma \nolimits_{1} + \mathop x\nolimits_{1} \mathop p\nolimits_{t - 1}^{{{\text{DD}}}} + \mathop u\nolimits_{2} \mathop q\nolimits_{t - 1}^{{{\text{RC}}}} + \mathop v\nolimits_{1} \mathop r\nolimits_{t - 1}^{{{\text{EU}}}} } \right.}}$$

4b $$P22_{t} = \frac{{\exp \left\{ {\mathop \gamma \nolimits_{2} + \mathop x\nolimits_{2} \mathop p\nolimits_{t - 1}^{{{\text{DD}}}} + \mathop u\nolimits_{2} \mathop q\nolimits_{t - 1}^{{{\text{RC}}}} + \mathop v\nolimits_{2} \mathop r\nolimits_{t - 1}^{{{\text{EU}}}} } \right.}}{{1 + \exp \left\{ {\mathop \gamma \nolimits_{2} + \mathop x\nolimits_{2} \mathop p\nolimits_{t - 1}^{{{\text{DD}}}} + \mathop u\nolimits_{2} \mathop q\nolimits_{t - 1}^{{{\text{RC}}}} + \mathop v\nolimits_{2} \mathop r\nolimits_{t - 1}^{{{\text{EU}}}} } \right.}}$$

From Eq. 4, the parameters $\mathop x\nolimits_{1}$ and $\mathop x\nolimits_{2}$, $\mathop u\nolimits_{1}$ and $\mathop u\nolimits_{2}$ in addition to $\mathop v\nolimits_{1}$ and $\mathop v\nolimits_{2}$, respectively, determine the significant impact of DD, RC, and the EU. The changes in DD, RC, and EU make the financial stress more likely to remain in state 1 (*stability*) and in state 2 (*unstable*/troubling) depending on the values of P11 and P22 in Eq. 4a, b. Other details regarding this approach are outlined in Hamilton (1989), but the estimation results and states’ probability diagrams are illustrated in Table 1 and Fig. 2, respectively.

**Fig. 3:**
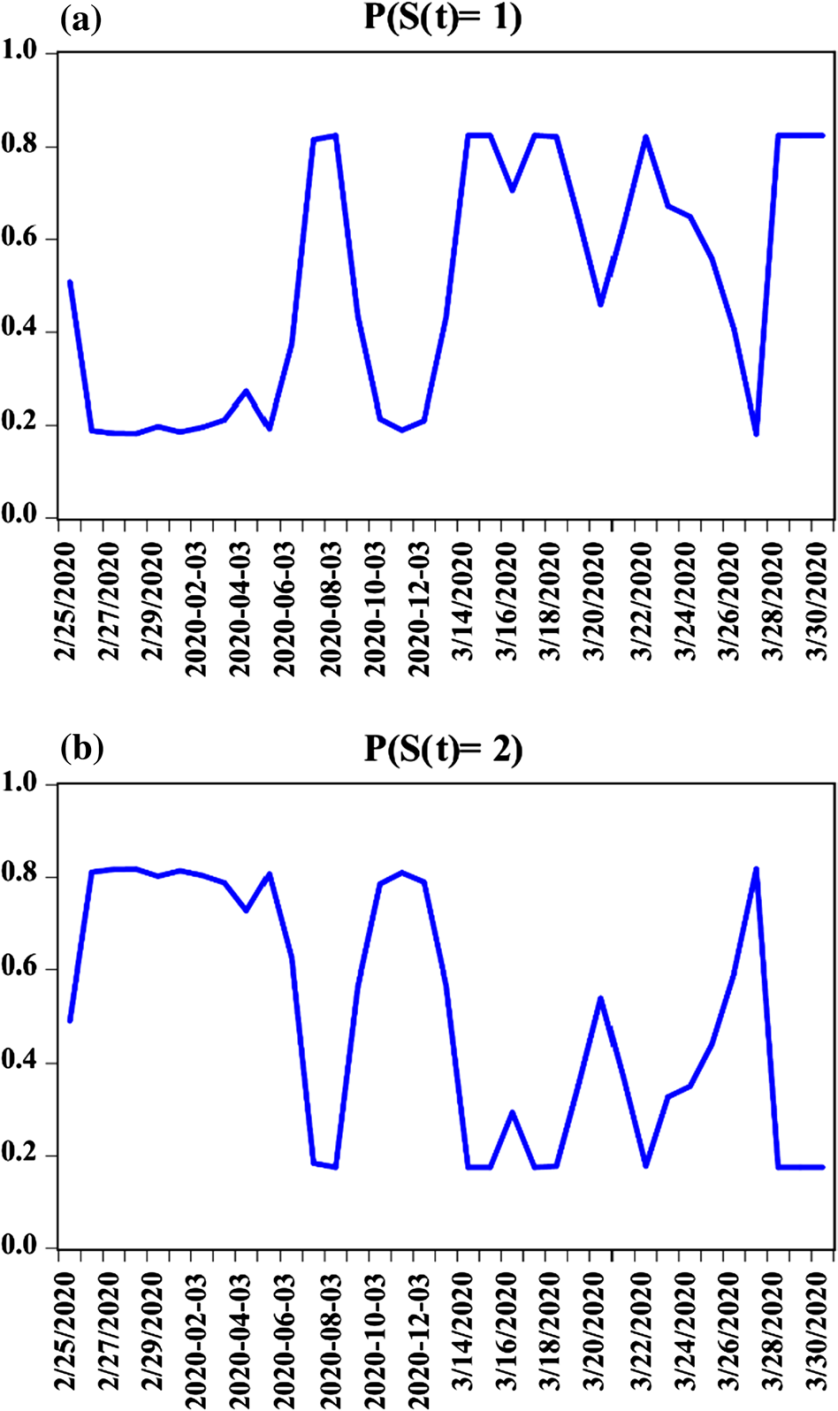
One-step ahead predicted regime probabilities: **a** duration dynamics and implied probabilities of State 1, **b** duration dynamics and implied probabilities of State 2

Additionally, to further establish the relationship between coronavirus and financial stress in the USA, the DD, RC, EU, and FS are further explored through the empirical models of FMOLS (fully modified ordinary least square, see Phillips and Hansen 1990), CCR (canonical cointegration regression, see Park 1992), and the ARDL (autoregressive distributed lag). The use of these methodologies, especially the ARDL (see Pesaran et al. 2001), is justified by the small sample size of the dataset. Also, the step-to-step of the methods are not highlighted in this study. However, by employing EVIEWs statistical software for the estimation, the series of output generated is summarized as results and diagnostics check are both presented in the lower part of Table 1 in addition to Fig. 3.

## Results and discussion

### Daily recoveries from COVID-19

Being a deadly disease and without a known cure or definite treatment, every recovery from COVID-19 is a significant scientific/medical achievement as well as a life-saving experience. With about 23% recovery rate from COVID-19 in the USA against the country’s about 6% death rate, the news of recoveries is certainly encouraging. Indicatively, the empirical results of both states (stable and distressing) financial stress in Table 1 show that the daily recoveries have a negative and significant impact of financial stress. This implies that a report of higher (increasing) recoveries from COVID-19 is responsible for minimal financial stress. Thus, the implication is that human lives are not only saved from the casualties of COVID-19, but the timely recovery of infected individuals also allays the potential burden of financial incapacitation of the sufferer and the vulnerable population. Moreover, the results from the FMOLS, CCR, and ARDL as implied in the lower part of Table 1 further affirm the desirable impact of recoveries on financial stress in the USA. Interestingly, this evidence confirms the statistical inference that put COVID-19 recovery rate as almost four (4) times the COVID-19 death rate in the USA.

### Daily deaths from COVID-19

With 89,564 American deaths from the COVID-19 pandemic, a new daily death record of 1394 as on 18 May 2020 implies that death rate from the number of confirmed total cases is a little above 6% (Johns Hopkins University and Medicine 2020). Previous work reported that an increase in confirmed cases of COVID-19 increases death rate by 0.2–0.8% daily (Owusu and Asumadu 2020). From the result, it is found that daily deaths from COVID-19 have not only been a source of bereavement to the Americans, but it has also remained a significant source of financial stress to the people. Implicatively, for every increase in the number of deaths per day by 1, financial stress increases by 0.006 and 0.012 in a more stable financial stress situation and a more distressing financial stress, respectively. Besides, the result from other estimation methods (the FMOLS, CCR, and ARDL) further complements the aforementioned evidence.

### Economic uncertainty amid COVID-19 pandemic

Similar to the impact of COVID-19 daily deaths on the Americans’ financial stress, the degree of uncertainty in the country’s economic policy is a significant determinant of financial stress. As seen in Table 3, for every unit increase in the level of economic uncertainty in the USA, there is a significant increase of 0.007 and 0.008 in the level of financial stress in a more stable state and distressing state, respectively. In a more assertive perspective, the results from the FMOLS, CCR, and ARDL estimation techniques further corroborate that economic uncertainty has a negative influence on financial stress in the USA. This illustrated evidence of economic uncertainty–financial stress nexus is closely supported by the previous studies that have hinted on the economic impact of infectious diseases (Keogh‐Brown et al. 2010; Nicola et al. 2020).

**Table 3:** States of financial stress: Evidence from Markov-Switch and cointegration techniques

Parameter	A (2 States) Markov-Switch evidence			
	State 1	State 2	Transition information	
	Coefficient	Coefficient	Probabilities	Durations
RC	− 0.002 [0.075^c^]	− 0.001 [0.000^a^]	State 1 → State 1 = 0.825	5.7 days
DD	0.006 [0.016^b^]	0.012 [0.000^a^]	State 1 → State 2 = 0.175	
EU	0.007 [0.000^a^]	0.008 [0.000^a^]	State 2 → State 2 = 0.810	5.5 days
C	0.483 [0.408]	− 1.103 [0.000^a^]	State 2 → State 1 = 0.181	
σ	− 0.516 [0.028^b^]	− 1.080 [0.000^a^]	–	–

	FMOLS	CCR	ARDL	ARDL Diagnostics
RC	− 0.002 [0.024^b^]	− 0.001 [0.455]	− 0.001 [0.278]	S: χ (*p*-value) = 0.389
DD	0.005 [0.010^b^]	0.003 [0.451]	0.001 [0.500]	H: χ (*p*-value) = 0.123
EU	0.010 [0.000^a^]	0.010 [0.000^a^]	0.003 [0.005^a^]	R^2^ = 0.95
C	− 0.974 [0.005^a^]	− 1.062 [0.004^a^]	− 0.176 [0.389]	*F*-statistic = 138.946^a^

*Note*: [.] is the probability; the varying factors FS, EU, DD, and RC are respectively the financial stress, economic uncertainty, daily deaths from coronavirus (COVID-19), and the daily recoveries from COVID-19 disease in the United States. Also, σ, C, R^2^, S, and H are respectively the standard deviation, intercept, *R*-Square, Breusch-Godfrey Serial Correlation Lagrange Multiplier test, and the Heteroskedasticity Breusch-Pagan-Godfrey test. The estimation shows that there is 1% (indicated as ^a^), 5% (indicated as ^b^), and 10% (indicated as ^c^) statistically significant correlation among the factors. FMOLS, CCR, and ARDL are the fully-modified ordinary least square, canonical cointegration regression, and autoregressive distributed lag techniques respectively

### Model validation

**Fig. 4:**
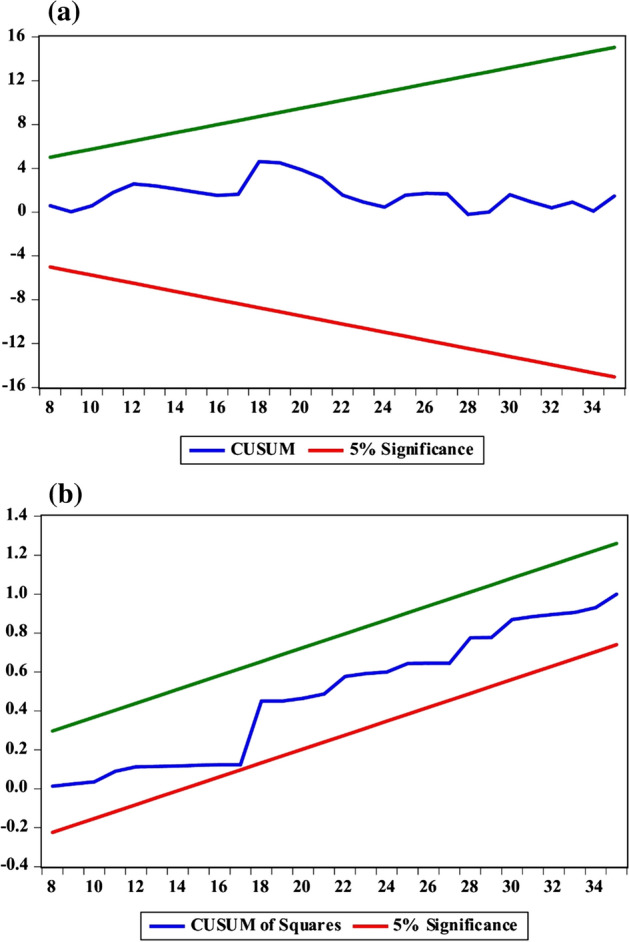
The stability evidence from the ARDL approach: **a** CUSUM, **b** CUSUM of Square

Several diagnostic checks were performed to further provide an assertive stance for the estimated n-COVID-19 model. Regarding the situations of the different (stable and distressing) states, the result shows that a change from more stable to distressing state is likely to happen about every 5.7 days while it takes about 5.5 days before transitioning from a more distressing to a stable state. Indicatively, there are 0.825 probability of having an enduring stable state and a 0.175 probability of switching from a stable state to distressing state. Similarly, a distressing state will endure with a probability of 0.810 and a 0.181 chance of transitioning from a distressing to a stable state (illustrated in Fig. 3). Importantly, giving the ARDL diagnostic results, the estimated model is free from experimental/statistical problems of heteroskedasticity and serial correlation (see Table 3) that could have compromised the result in addition to the evidence of stability from Fig. 4.

## Conclusion

Until now, and since the end of the Great Depression of the 1930s, the USA has not experienced the level of economic devastation arising from the nCOVID-19 pandemic. With the current speculated unemployment rate heading to 20% and two stimulus packages already handed out to the Americans, the impact of the virulent disease on the USA’ economic activities and financial market cannot be less devastating. In this context, the current study examined how the daily deaths and daily recoveries from COVID-19 in addition to the uncertainty in the US economic policy have impacted the Americans’ financial stress in a different dimension. The startling finding revealed that COVID-19 daily recoveries will cause a significant relief to the US health care system and decline potential pressure on the financial and socio-economic means of the Americans. However, daily deaths from COVID-19 pandemic, reproductive rate of COVID-19 cases and uncertainty of government’s economic policy are two significant factors with the potential of plunging the USA into a state of financial stress. This study revealed the existence of two states of* stable* and more* distressing* financial stress with a certainty of 0.825 (lasting 5.7 days) and 0.810 (lasting 5.5 days).

Importantly, the result of this investigation has not provided an interesting perspective without an apparent policy direction. Foremost, considering that no known cure has been proffered for COVID-19 yet, a more concerted effort should be directed at improving the treatment of infected patients to overwhelming increase the recovery rate of COVID-19 sufferers. Thus, by increasing the recovery rate, the financial stress among Americans will be significantly mitigated. Moreso, the direction of the country’s current economic policy can be further reviewed in the context of the existing COVID-19 pandemic such that a more workable country-specific policy that is capable of further minimizing the Americans’ financial stress is deployed retrospectively. Moreover, future study could consider the impact of COVID-19 across racial divide, especially in the context of regime switching.

## Data Availability

Data for analysis in this study are included in this published article.

## Footnotes

1 More information on EPU is available at https://www.policyuncertainty.com/.
